# High-grade appendiceal mucinous neoplasms in children: a case report

**DOI:** 10.3389/fped.2025.1525530

**Published:** 2025-04-24

**Authors:** Hongli Wang, Jin Zhang, Xiwei Hao, Hongting Lu, FuJiang Li, Cong Shang

**Affiliations:** ^1^ Department of Pediatric Surgery, The Affiliated Hospital of Qingdao University, Qingdao, China; ^2^ Department of Pediatric Respiratory Medicine, Qingdao Women and Children's Hospital, Qingdao, China; ^3^ Department of Pediatric Surgery, Qingdao Women and Children’s Hospital, Qingdao, China

**Keywords:** appendiceal mucinous neoplasms, appendicitis, pediatrics, surgery, case report

## Abstract

**Background:**

Appendiceal Mucinous Neoplasms (AMNs) are a rare disease characterized by the accumulation of mucus within the vermiform appendix and are frequently misdiagnosed as appendicitis. Hence, it is crucial to consider AMNs because they have the potential to progress into peritoneal pseudomyxoma (PP), a clinical syndrome distinguished by mucus buildup in the peritoneum leading to progressive abdominal pathology.

**Case report:**

We present a case involving a 13-year-old male patient who was initially suspected of having acute purulent appendicitis prior to surgery, a formal laparoscopic appendectomy was performed. Microscopic examination revealed the presence of high-grade appendiceal mucinous neoplasm (HAMN), with certain areas exhibiting features consistent with mucinous adenocarcinoma, and focal invasion of the muscular layer was observed. After multidisciplinary discussion, the patient underwent laparoscopic ileocecal resection followed by hyperthermic intraperitoneal chemotherapy (HIPEC) and molecular targeted therapy leading to favorable outcome during subsequent regular follow-up evaluations validating the appropriateness of the chosen surgical procedure.

**Conclusion:**

This case presents a rare pediatric appendiceal mucinous tumor, highlighting the importance of recognizing the presence of a tumor for clinicians when diagnosing an appendiceal abscess.

## Introduction

The incidence of Appendiceal Mucinous Neoplasms (AMNs) among appendiceal tumors was reported to be 1.4% (1). The predominant symptom observed in patients with AMNs was right lower quadrant pain, and the median age at diagnosis was 60 ± 15 years (1). Furthermore, there was a notable predominance of females (1, 2). Appendiceal mucinous neoplasms are often confused with appendicitis due to the prevalent symptom of right lower quadrant abdominal pain (1, 3). It is crucial to consider the possibility of AMNs in the differential diagnosis of abdominal pain since these neoplasms can be diagnosed at any age (3). Although there are established pathological classifications, surgical resection is vital in preventing the progression of peritoneal pseudomyxoma (PP) (1, 3–8). However, preoperative diagnosis of AMNs poses significant challenge due to its nonspecific clinical manifestations.

Our goal is to enhance awareness of AMNs in the pediatric population and contribute to the existing knowledge by sharing our experiences with a case treated at our hospital, by providing information on classification and treatment options for AMNs.

## Case presentation

A 13-year-old male patient was presented to our hospital with a complaint of right lower quadrant abdominal pain and constipation for 20 days, accompanied by a weight loss of 5 kg. One day prior to his visit to the pediatric surgery department, he developed fever up to 38.9 °C. At his local hospital, an initial diagnosis of periappendiceal abscess was made based on computed tomography (CT) findings. Upon abdominal examination conducted at our hospital, tenderness was noted in the right lower quadrant, with no signs of abdominal tension or rebound tenderness.

**Figure 1:**
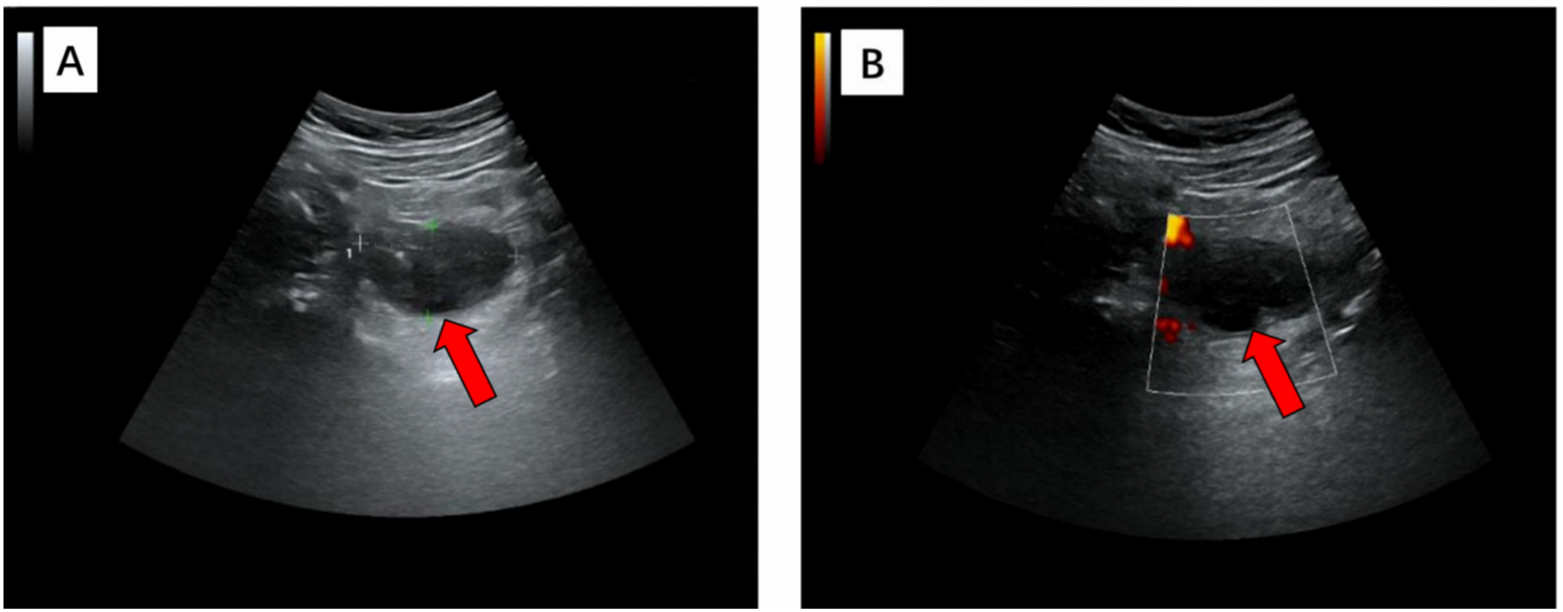
In grayscale **(A)** and color Doppler **(B)** longitudinal ultrasound images, a heterogeneous hypoechoic mass with a maximum diameter of 5.4 cm was observed, exhibiting clear margins and an irregular outline. Further examination with color Doppler imaging revealed the absence of no significant internal blood flow, thereby confirming its cystic nature.

**Figure 2:**
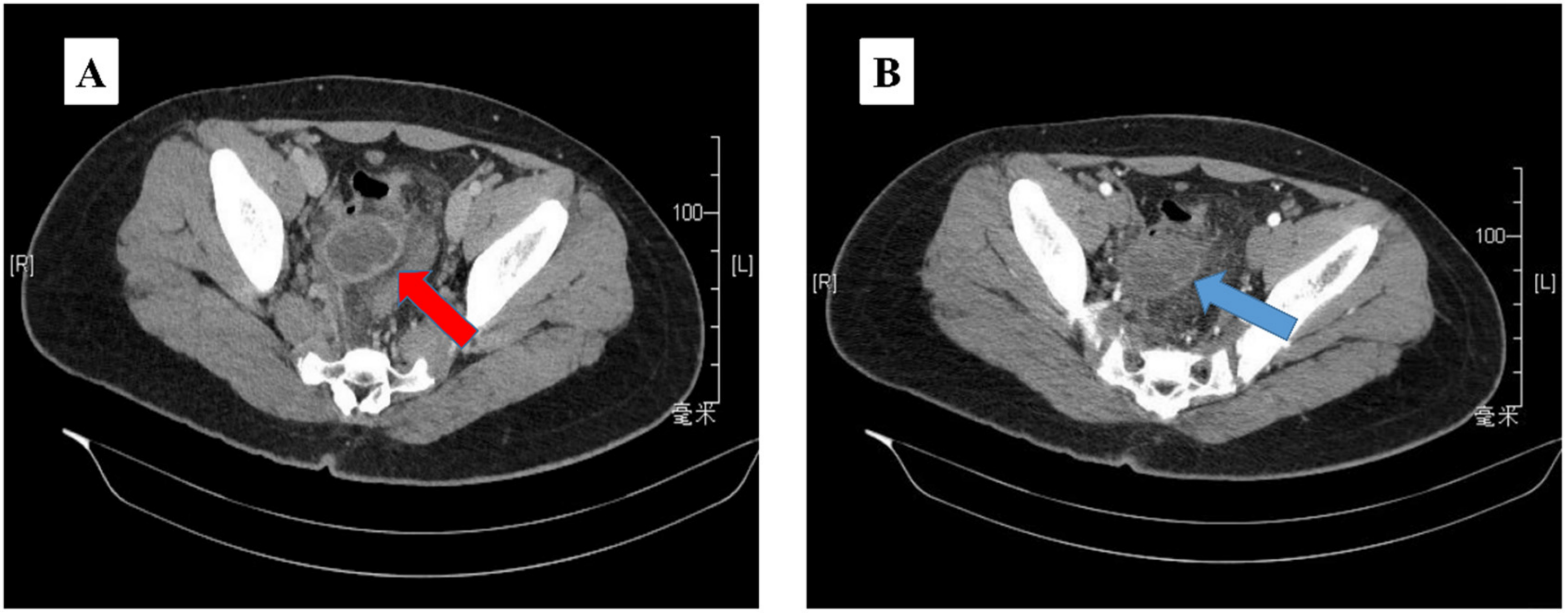
The abdominopelvic contrast-enhanced CT **(A)** revealed a mixed density mass 37 mm × 44 mm, predominantly with cystic density and a few soft tissue density shadows in between (red arrow). The mass exhibited clear delayed enhancement **(B)** during the enhanced scan (blue arrow).

**Figure 3:**
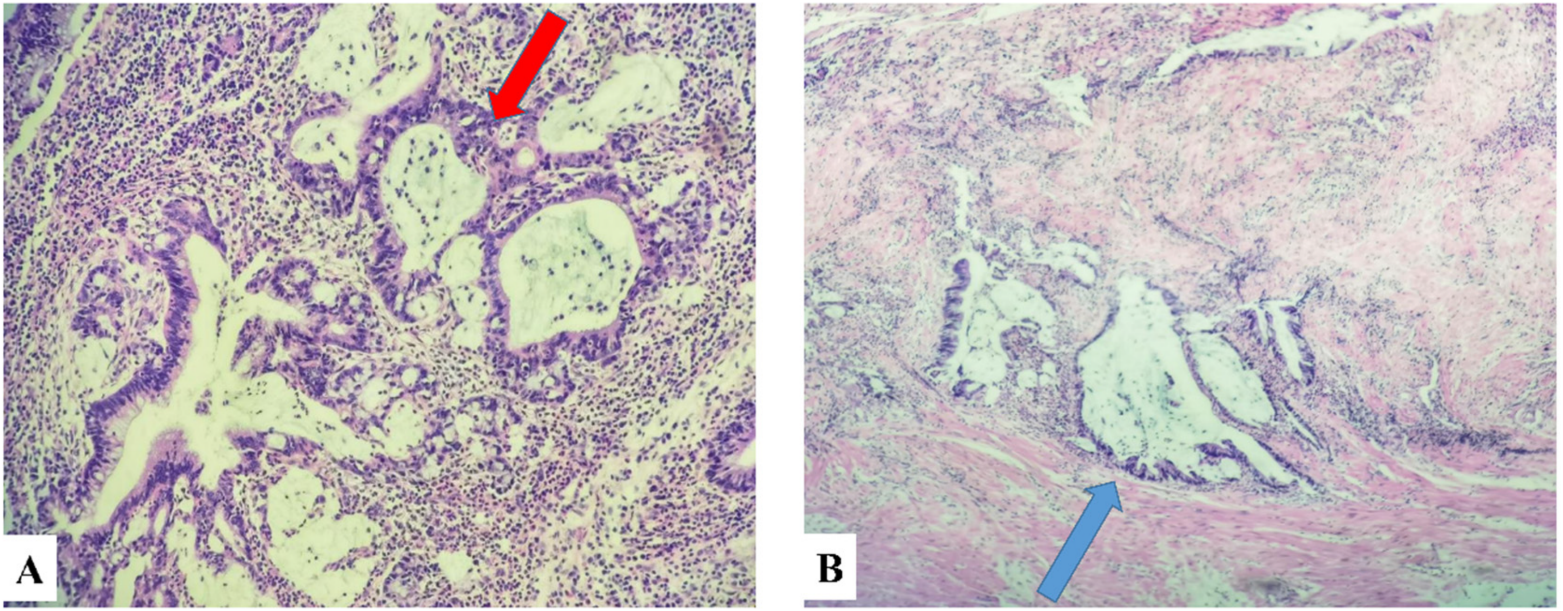
Panel **(A)** was pathologically diagnosed as HAMN of the appendix (red arrow), and panel **(B)** was characterized by partial regional involvement of mucinous adenocarcinoma (black arrow).

The ultrasound examination revealed the presence of a heterogeneous and hypoechoic mass measuring approximately 54.0 mm × 32.0 mm × 34.0 mm with well-defined borders located at the tip of the appendix (Figure 1). Notably, no significant intralesional color Doppler flow was observed (Figure 1B). Further diagnostic information was obtained through an enhanced CT scan at our hospital, which revealed a mixed-density mass in the appendiceal region measuring about 37.0 mm × 44.0 mm in cross-section. The mass predominantly exhibited cystic density with a few soft tissue density shadows present, while the soft tissue density demonstrated noticeable delayed enhancement along with evidence of a periappendicular abscess involvement of the adjacent sigmoid colon (Figure 2). Blood testing results showed C—reactive protein (CRP) levels of 58.58 mg/L, carcinoembryonic antigen (CEA) levels of 7.99 ng/ml and neuron-specific enolase (NSE) levels of 168.7 ng/ml; other results were generally normal. The patient underwent a laparoscopic procedure during which an abscess was fund, between the bladder and rectum, which wrapped around the tip of the appendix, purulent fluid was attached to the appendix; a presumed diagnosis of perforated appendicitis was made. Cultures were obtained and the appendix removed. The patient exhibited a satisfactory postoperative recovery, and the presence of Actinomyces odontolyticus infection was confirmed through bacterial culture. The postoperative pathological examination revealed the coexistence of high-grade appendiceal mucinous neoplasm (HAMN) with partial mucinous adenocarcinoma and focal invasion into the muscular layer (Figure 3).

Postoperative positron emission tomography (PET/CT) revealed multiple enlarged lymph nodes of uncertain nature in the abdominal cavity. Due to the risk of mucinous adenocarcinoma rupture and progress to PP, the patient underwent a laparoscopic ileoceal resection and received three consecutive sessions of hyperthermic intraperitoneal chemotherapy (HIPEC) three months after surgery. The first session involved a 60-min intraperitoneal perfusion with distilled water at 43 ℃, the second session received a subsequent 90-min infusion of raltitrexed at a dose of 4 mg/m^2^. Finally, the third session consisted of a 90-min intraperitoneal perfusion with cisplatin at a dose of 100 mg. The patient had an uneventful recovery and was discharged from the hospital. Pathological examination revealed negative resection margins and no regional lymph nodes metastasis. Follow-up observations every three months was recommended. At 16 months after surgery, an enhanced CT scan showed a new mass invading the right ureter and closely related to both iliac vessels and bowel in the pelvis area, posing challenges for radical resection. Following comprehensive evaluation, FOLFIRI (fluorouracil, leucovorin, irinotecan) chemotherapy regimen + bevacizumab targeted therapy was initiated as treatment. The medical team advised serial CT scans every 3 months along with regular physical examinations for follow-up observations. At the last follow-up, which occurred 19 months after the operation, he remained well; however, the patient chose to continue follow-up care at another institution.

## Discussion

Appendiceal tumors are found in approximately 1% of appendectomy specimens (2). The incidence of AMNs among appendiceal tumors is reported to be 1.4% (1). Frequently, AMNs are misdiagnosed as cystic masses, adnexal abscesses, pedunculated uterine leiomyoma or appendiceal mucoceles, primarily due to their most common presenting symptom: acute or chronic right lower quadrant abdominal pain (9–13). There have been documented cases where AMNs coexist with other medical conditions; for example, Propst R. reported a case of metastatic prostate adenocarcinoma coexisting with a history of an HAMN (14).

The eighth edition of the American Joint Committee on Cancer (AJCC) Staging Manual has introduced a three-tiered system: G1 designates low-grade tumors, while G2 and G3 classify high-grade tumors (6). The Peritoneal Surface Oncology Group International (PSOGI) has defined Low-grade appendiceal mucinous neoplasm (LAMN) as a mucinous neoplasm exhibiting low-grade cytology, which may manifest with any of the subsequent characteristics (5, 15, 16): absence of the lamina propria and muscularis mucosae; fibrosis occurring in the submucosa; a growth pattern characterized by invagination into the appendix wall. In terms of gross features and histology, HAMN exhibits similarities to LAMN (5, 12), however, HAMN differs in that it extends beyond the mucosa into the appendiceal wall with high-grade cytologic atypia (12).

It has been suggested that patients with HAMNs should refer to the AJCC staging system for LAMN (12). However, PSOGI and the 8th edition of AJCC recommend applying the invasive adenocarcinoma staging system to HAMN (17). Therefore, further investigations are needed to establish a standardized staging criterion for HAMN.

The treatment approach for HAMN is determined by the tumor's stage and histological features, typically involving adjuvant right hemicolectomy, cytoreductive surgery (CRS), and HIPEC, preoperative chemotherapy or targeted therapy may also be utilized in some cases (17, 18). A clinical trial conducted by Raul S. Gonzalez demonstrated that 2 out of 35 HAMN patients who underwent appendectomy had positive proximal margins but remained alive and recurrence-free during follow-up. This finding suggests that when mucinous neoplasms are confined to the appendix (12), appendectomy alone may be a viable option. However, according to PSOGI recommendations, adjunctive right hemicolectomy should be considered for HAMN patients without perforation, whereas perforation after appendectomy requires right hemicolectomy and CRS + HIPEC. For cases of appendiceal mucinous adenocarcinoma, right hemicolectomy followed by CRS + HIPEC is recommended (15, 17, 19). Additionally, some experts suggest considering preoperative systemic chemotherapy for patients with high-grade appendiceal adenocarcinoma (6, 20).

The necessity of extended resection following appendectomy has been a subject of debate among researchers. Routine right hemicolectomy is not recommended in patients with negative appendiceal margins (2, 5, 17, 21) due to the unlikely spread of tumor cells to regional lymph nodes and the potential presence of extra-appendiceal cells only within scar tissue (5, 18). Some investigators have suggested that non-LAMN appendiceal cancers invading beyond the submucosa may require additional resection approaches similar to those employed for colorectal cancer (22). However, given the limited published data on appendiceal neoplasms, further prospective studies are warranted to determine the optimal treatment regimen.

## Conclusion

This case reports a rare case of appendiceal mucinous neoplasm in children, emphasizing the importance for pediatric surgeons to consider the potential presence of AMNs when diagnosing appendiceal masses.

## Data Availability

The raw data supporting the conclusions of this article will be made available by the authors, without undue reservation.
